# Evaluation of antioxidant enzyme activity among Indian patients with type 2 diabetes mellitus

**DOI:** 10.6026/973206300215029

**Published:** 2025-12-15

**Authors:** Ritambhara Singh, Hemali Jha, Geetika Gupta, Amrit Podder, Parth Jani, Kanchan Sonone

**Affiliations:** 1 Department of Physiology, Santosh Medical College, Ghaziabad, Uttar Pradesh, India; 2 Department of Internal Medicine, Integral Institute of Medical Sciences and Research, Integral University, Lucknow, Uttar Pradesh, India; 3 Department of Physiology, Acharya Shri Chander College of Medical Sciences & Hospital, Sidhra, Jammu, India; 4 Department of Physiology, Teerthanker Mahaveer Medical College & Research Centre, Teerthanker Mahaveer University, Moradabad, Uttar Pradesh, India; 5 Department of General Medicine, Government Medical College, Bhavnagar, India; 6 Department of Biochemistry, SMBT IMSRC, Nashik, Maharashtra, India

**Keywords:** Antioxidant enzymes, catalase, glutathione peroxidase, oxidative stress, type 2 diabetes

## Abstract

Oxidative stress is a key factor in the pathogenesis of Type 2 Diabetes Mellitus (T2DM), and the role of antioxidant enzymes in managing this stress remains an important area of research. Therefore, it is of interest to investigate the activity of antioxidant enzymes (SOD, CAT and GPx) in patients with Type 2 Diabetes Mellitus (T2DM) to understand the relationship between glycemic control and oxidative stress. We found significantly lower enzyme activity in individuals with poor glycemic control (HbA1c ≥ 7%) compared to those with better control (HbA1c < 7%). Negative correlations were observed between antioxidant enzyme levels and clinical variables such as HbA1c and fasting blood glucose. Our findings suggest that antioxidant enzyme activity may play a crucial role in managing oxidative stress in T2DM. Further studies should explore the potential of antioxidant-based therapies. The advancement to knowledge in this study is the identification of reduced antioxidant enzyme activity in patients with poor glycemic control, highlighting its potential role in managing oxidative stress in Type 2 Diabetes Mellitus.

## Background:

Type 2 diabetes mellitus (T2DM) is a prevalent metabolic disorder characterized by insulin resistance and impaired glucose metabolism. The condition is associated with numerous complications, including cardiovascular diseases, kidney dysfunction and neuropathy, which significantly affect the quality of life of individuals [1]. In addition to the well-established risk factors of obesity, physical inactivity and genetic predisposition, recent research has emphasized the role of oxidative stress in the pathogenesis of T2DM [2]. Oxidative stress occurs when there is an imbalance between the production of reactive oxygen species (ROS) and the body's ability to neutralize them with antioxidants. This imbalance contributes to cellular damage, inflammation and insulin resistance, all of which play pivotal roles in the development and progression of diabetes and its complications [3]. Antioxidant enzymes are essential in the body's defense against oxidative stress. These enzymes include superoxide dismutase (SOD), catalase (CAT) and glutathione peroxidase (GPx), which help neutralize ROS and protect cells from oxidative damage [4]. However, studies have shown that individuals with T2DM often exhibit decreased antioxidant enzyme activity, leading to a greater accumulation of ROS and increased oxidative damage. This reduction in antioxidant capacity is thought to exacerbate insulin resistance, impair pancreatic β-cell function and accelerate the development of diabetic complications [5]. Numerous studies have found that patients with T2DM had significantly lower levels of SOD and CAT compared to healthy controls [6]. Another study highlighted that chronic hyperglycemia could induce oxidative stress and reduce the activity of key antioxidant enzymes. The reduction in antioxidant defense mechanisms in T2DM has been linked to endothelial dysfunction, increased vascular permeability and the promotion of inflammatory processes that contribute to diabetic complications, particularly cardiovascular disease and nephropathy [7]. While several studies have evaluated the antioxidant status in T2DM patients, results have been inconsistent, possibly due to differences in study design, sample size and ethnic diversity [8]. Furthermore, the effects of pharmacological interventions and lifestyle changes, such as diet and exercise, on antioxidant enzyme activity in patients with diabetes remain underexplored. Therefore, understanding the status of antioxidant enzyme activity in patients with T2DM and its correlation with disease progression is crucial for developing effective strategies to manage and prevent diabetic complications [9]. Therefore, it is of interest to determine the levels of antioxidant enzyme activity in patients with Type 2 Diabetes Mellitus and their association with disease severity, providing insights into potential therapeutic approaches for managing oxidative stress in diabetes.

##  Methodology:

This cross-sectional study aimed to evaluate the antioxidant enzyme activity in 80 patients diagnosed with Type 2 Diabetes Mellitus (T2DM). Participants were selected based on their diagnosis of T2DM for at least one year, aged between 40 and 70 years and were either on oral anti-diabetic medications or insulin therapy. Individuals with other chronic conditions such as cardiovascular diseases, renal failure, or active infections were excluded to avoid confounding factors. The study was approved by the Institutional Review Board (IRB) of the participating hospital and all participants provided informed consent. Upon enrollment, participants underwent a clinical evaluation that included medical history, anthropometric measurements (weight, height and body mass index) and blood pressure measurements. Laboratory tests were conducted to assess fasting blood glucose (FBG) and HbA1c levels, key indicators of glycemic control. Blood samples were collected after an overnight fast of 10-12 hours from the antecubital vein and 5 mL of blood was drawn using sterile techniques. The blood was processed immediately to separate plasma and red blood cells and serum samples were stored at -80°C for subsequent analysis of antioxidant enzyme activity. The activity of three major antioxidant enzymes-superoxide dismutase (SOD), catalase (CAT) and glutathione peroxidase (GPx)-was measured in the serum samples using well-established biochemical methods. SOD activity was determined by its ability to inhibit the reduction of nitroblue tetrazolium (NBT) in a colorimetric assay, CAT activity was assessed by the rate of hydrogen peroxide (H_2_O_2_) degradation measured spectrophotometrically and GPx activity was measured through a coupled reaction with glutathione and hydrogen peroxide, with the reduction of NADPH monitored spectrophotometrically. Descriptive statistics were used to summarize demographic and clinical characteristics of the participants, with the mean ±standard deviation (SD) for continuous variables and frequencies for categorical variables. To compare antioxidant enzyme activity between participants with good (HbA1c < 7%) and poor (HbA1c ≥ 7%) glycemic control, an independent t-test was applied. Correlations between antioxidant enzyme activity and clinical variables, including age, BMI, FBG and HbA1c, were assessed using Pearson's correlation coefficient. A p-value of <0.05 was considered statistically significant and all analyses were performed using SPSS software version 25.0 (IBM Corp, Armonk, NY). This study, while providing valuable insights into the antioxidant enzyme activity in T2DM patients, is limited by its cross-sectional design, which does not establish causal relationships. Additionally, lifestyle factors such as diet and physical activity were not assessed in detail and future studies should consider longitudinal designs and comprehensive lifestyle evaluations to explore these relationships further.

## Results:

A total of 80 participants (45 males and 35 females) with Type 2 Diabetes Mellitus (T2DM) were included in this study. Their demographic characteristics, including age, body mass index (BMI), fasting blood glucose (FBG) and HbA1c levels, were recorded and analyzed to determine the relationship between these factors and antioxidant enzyme activity. The study also examined the activity of three antioxidant enzymes: superoxide dismutase (SOD), catalase (CAT) and glutathione peroxidase (GPx) in the serum samples of the participants. Descriptive statistics, t-tests and Pearson's correlation coefficients were used to assess the relationship between these variables. The mean age of participants was 58 years, with a BMI of 29.4 kg/m^2^, indicating that the majority were overweight or obese. The average fasting blood glucose (FBG) level was 172 mg/dL and HbA1c was 8.4%, suggesting suboptimal glycemic control in most participants. Blood pressure measurements showed that the participants had mild to moderate hypertension, with an average systolic blood pressure of 130 mmHg and diastolic blood pressure of 85 mmHg (Table 1 - see PDF). Antioxidant enzyme activity was significantly lower in participants with poor glycemic control (HbA1c ≥ 7%) compared to those with good glycemic control (HbA1c < 7%). Specifically, SOD, CAT and GPx activities were significantly reduced in the poor glycemic control group (p < 0.05 for all comparisons) (Table 2 - see PDF). Significant negative correlations were found between antioxidant enzyme activity and clinical variables such as age, BMI, fasting blood glucose and HbA1c levels. The most significant correlations were observed between HbA1c levels and antioxidant enzyme activities, particularly SOD (r = -0.42), indicating that higher HbA1c levels were associated with lower antioxidant enzyme activity (Table 3 - see PDF). There were no significant differences in antioxidant enzyme activity between male and female participants (p > 0.05 for all comparisons), suggesting that gender did not influence the antioxidant enzyme activity in this cohort (Table 4 - see PDF). There was a moderate negative correlation between SOD and systolic blood pressure (r = -0.33, p = 0.05), indicating that higher antioxidant enzyme activity was associated with lower systolic blood pressure. The correlation was weaker for diastolic blood pressure but still negative, suggesting that antioxidant activity may be linked to blood pressure regulation in T2DM patients (Table 5 - see PDF).

The participants in this study exhibited an average age of 58 years, with most falling within the overweight category (BMI = 29.4). The majority of participants had suboptimal glycemic control, as evidenced by an average HbA1c of 8.4%. Blood pressure measurements indicated mild to moderate hypertension. In terms of antioxidant enzyme activity, significant reductions in SOD, CAT and GPx levels were observed in participants with poor glycemic control (HbA1c ≥ 7%), suggesting that impaired glycemic control leads to diminished antioxidant defense. Moreover, negative correlations between antioxidant enzyme activity and clinical variables such as HbA1c and fasting blood glucose levels further support the hypothesis that oxidative stress is exacerbated by poor glycemic control. No significant differences were found between male and female participants. A moderate negative correlation was observed between SOD activity and systolic blood pressure, highlighting the potential role of antioxidants in blood pressure regulation. These results are consistent with previous studies, which have shown that oxidative stress plays a key role in the pathophysiology of T2DM. They suggest that maintaining antioxidant enzyme activity may be important in managing diabetes and its complications.

## Discussion:

This study aimed to evaluate the activity of antioxidant enzymes-superoxide dismutase (SOD), catalase (CAT) and glutathione peroxidase (GPx) in patients with Type 2 Diabetes Mellitus (T2DM). The findings revealed significantly reduced enzyme activities in individuals with poor glycemic control (HbA1c ≥ 7%) compared to those with better control (HbA1c < 7%). These results align with previous research highlighting the role of oxidative stress in T2DM pathogenesis. A study by Dworzanski *et al.* (2020) [10] reported decreased SOD and GPx activities in T2DM patients, particularly in those with prolonged disease duration and obesity. Our study corroborates these findings, demonstrating that poor glycemic control is associated with diminished antioxidant enzyme activity. Furthermore, our research observed a moderate negative correlation between SOD activity and systolic blood pressure, suggesting that antioxidant enzyme levels may influence blood pressure regulation in T2DM patients. In contrast, a study by Cecerska-Heryc *et al.* (2025) [11] found elevated SOD activity in T2DM patients compared to healthy controls, which they attributed to a compensatory response to increased oxidative stress. This discrepancy may be due to differences in study design, sample size and patient characteristics. Our study's consistent findings across various clinical variables strengthen the argument that reduced antioxidant enzyme activity is a hallmark of poor glycemic control in T2DM. Additionally, the study by Arias-Chavez *et al.* (2023) [12] found that lower SOD and CAT activities were associated with an increased risk of T2DM, highlighting the importance of antioxidant defenses in diabetes prevention. Our study extends this understanding by linking reduced antioxidant enzyme activity to existing diabetic complications, highlighting the need for antioxidant-based therapeutic strategies. While our study provides valuable insights into the relationship between antioxidant enzyme activity and glycemic control in type 2 diabetes mellitus (T2DM), it is not without limitations. The cross-sectional design precludes causal inferences and the lack of assessment of lifestyle factors such as diet and physical activity may have influenced the results. Future longitudinal studies incorporating these variables are necessary to elucidate further the role of antioxidant enzymes in T2DM progression and management.

## Conclusion:

We show the reduced activity of antioxidant enzymes in patients with poor glycemic control, emphasizing the role of oxidative stress in Type 2 Diabetes Mellitus. The findings suggest that maintaining antioxidant enzyme activity could help manage oxidative damage in diabetes. Future research should explore antioxidant-based therapies to improve disease outcomes.
